# Use of Machine-Learning Algorithms in Intensified Preoperative Therapy of Pancreatic Cancer to Predict Individual Risk of Relapse

**DOI:** 10.3390/cancers11050606

**Published:** 2019-04-30

**Authors:** Pablo Sala Elarre, Esther Oyaga-Iriarte, Kenneth H. Yu, Vicky Baudin, Leire Arbea Moreno, Omar Carranza, Ana Chopitea Ortega, Mariano Ponz-Sarvise, Luis D. Mejías Sosa, Fernando Rotellar Sastre, Blanca Larrea Leoz, Yohana Iragorri Barberena, Jose C. Subtil Iñigo, Alberto Benito Boíllos, Fernando Pardo, Javier Rodríguez Rodríguez

**Affiliations:** 1 Department of Medical Oncology, Clínica Universidad de Navarra, Pamplona, 31008 Navarra, Spain; psala@unav.es (P.S.E.); ocarranza@hotmail.com (O.C.); chopitea@unav.es (A.C.O.); mponz@unav.es (M.P.-S.); yiragorri@unav.es (Y.I.B.); 2 Department of Mathematics and Statistics, Pharmamodelling, Noain, 31110 Navarra, Spain; eoyaga@pharmamodelling.es; 3 Gastrointestinal Oncology Service, Memorial Sloan Kettering Cancer Center, New York, NY 10065, USA; YuK1@mskcc.org; 4 Weill Cornell Medical College, New York, NY 10065, USA; 5 Human Oncology and Pathogenesis Program, Collaborative Research Centers, Memorial Sloan Kettering Cancer Center, New York, NY 10065, USA; baudinv@mskcc.org; 6 Department of Radiation Oncology, Clínica Universidad de Navarra, Pamplona, 31008 Navarra, Spain; larbea@unav.es; 7 Department of Pathology, Hospital Universitario Rey Juan Carlos, Móstoles, 28933 Madrid, Spain; luis.mejiass@hospitalreyjuancarlos.es; 8 Department of HPB Surgery, Clínica Universidad de Navarra, Pamplona, 31008 Navarra, Spain; frotellar@unav.es (F.R.S.); blarreal@unav.es (B.L.L.); 9 Department of Gastroenterology, Clínica Universidad de Navarra, Pamplona, 31008 Navarra, Spain; jcsubtil@unav.es; 10 Department of Radiology, Clínica Universidad de Navarra, Pamplona, 31008 Navarra, Spain; albenitob@unav.es

**Keywords:** pancreatic, resectable, neoadjuvant chemotherapy, neoadjuvant chemoradiation, machine-learning, model-based prediction

## Abstract

Background: Although surgical resection is the only potentially curative treatment for pancreatic cancer (PC), long-term outcomes of this treatment remain poor. The aim of this study is to describe the feasibility of a neoadjuvant treatment with induction polychemotherapy (IPCT) followed by chemoradiation (CRT) in resectable PC, and to develop a machine-learning algorithm to predict risk of relapse. Methods: Forty patients with resectable PC treated in our institution with IPCT (based on mFOLFOXIRI, GEMOX or GEMOXEL) followed by CRT (50 Gy and concurrent Capecitabine) were retrospectively analyzed. Additionally, clinical, pathological and analytical data were collected in order to perform a 2-year relapse-risk predictive population model using machine-learning techniques. Results: A R0 resection was achieved in 90% of the patients. After a median follow-up of 33.5 months, median progression-free survival (PFS) was 18 months and median overall survival (OS) was 39 months. The 3 and 5-year actuarial PFS were 43.8% and 32.3%, respectively. The 3 and 5-year actuarial OS were 51.5% and 34.8%, respectively. Forty-percent of grade 3-4 IPCT toxicity, and 29.7% of grade 3 CRT toxicity were reported. Considering the use of granulocyte colony-stimulating factors, the number of resected lymph nodes, the presence of perineural invasion and the surgical margin status, a logistic regression algorithm predicted the individual 2-year relapse-risk with an accuracy of 0.71 (95% confidence interval [CI] 0.56–0.84, *p* = 0.005). The model-predicted outcome matched 64% of the observed outcomes in an external dataset. Conclusion: An intensified multimodal neoadjuvant approach (IPCT + CRT) in resectable PC is feasible, with an encouraging long-term outcome. Machine-learning algorithms might be a useful tool to predict individual risk of relapse. A small sample size and therapy heterogeneity remain as potential limitations.

## 1. Introduction

Pancreatic cancer (PC) is expected to be the second cause of cancer deaths in Western countries by 2030. [1,2] Surgical resection remains the only potentially curative treatment, but only 10–20% of cases are resectable at diagnosis. Even in this setting, long-term outcomes remain dismal, with a 5-year overall survival rate of 10%. [3] This poor prognosis has led to the use of multimodal approaches, including adjuvant chemotherapy [3,4,5,6,7,8], with a clear impact on 5-year overall survival, [6] and adjuvant chemoradiation just in case of microscopically positive margins and/or lymph node involvement [5,9,10,11].

The high rate of disease relapse, coupled with a low compliance (only 51% of patients outside the context of a clinical trial receive adjuvant therapy [12]) remain major drawbacks of an adjuvant strategy. This has led some authors to uphold the use of preoperative treatment, with potential advantages such as an increased R0 resection rate, better compliance, a reduction in the risk of intraoperative tumor spillage and avoidance of unnecessary surgery, with its related morbidity and mortality, in patients with unfavorable tumor biology. Preliminary data with this approach, either preoperative chemotherapy or chemoradiation, seem promising. [13,14]

The development of predictive tools for individual relapse-risk assessment after upfront multimodal therapy may help to further optimize treatment decision-making. While nomograms [15] and molecular prognostic signatures have been validated in other solid tumors, their role in PC seems limited. [16,17,18] The use of population models and machine learning algorithms to predict disease evolution might be considered as a potential alternative for this tumor type. [19,20,21,22,23,24,25,26]

The aim of the present retrospective analysis is two-fold. First, to describe the clinical results achieved with the use of induction polychemotherapy (IPCT) followed by chemoradiation (CRT) in patients with resectable PC. Second, to evaluate the individual 2-year relapse-risk on a supervised machine-learning algorithm basis, among a group of patients with PC resected after preoperative therapy.

## 2. Materials and Methods

### 2.1. Patient Elegibility

All patients diagnosed of potentially curable PC from September 2005 to November 2016 were evaluated by a multidisciplinary team composed of hepatobiliary surgeons, endoscopists, interventional radiologists, medical and radiation oncologists. Initial workup included: clinical examination, laboratory tests including a serum CA-19.9 level, endoscopic ultrasound (EUS) with guided fine needle aspiration biopsy (FNA) of the pancreatic lesion and a CT-scan to define the extent of the disease. When needed, biliary decompression was performed with an endobiliary stent prior to neoadjuvant treatment. Staging laparoscopy was considered in stage IV suspicious cases.

Patients were considered for neoadjuvant therapy if they had a good performance status (≤2 according to Eastern Cooperative Oncology Group (ECOG)), an adequate hematological, renal and liver function, and a histologically confirmed resectable/borderline-resectable PC. The criteria applied to determine resectability were based on the National Comprehensive Cancer Network, according to the consensus statement of the Society of Abdominal Radiology and the American Pancreatic Association (Table S1).

### 2.2. Neoadjuvant Therapy

Neoadjuvant treatment consisted of 2–4 months of IPCT followed, in the case of stable or responding disease, by CRT.

Most patients received IPCT on an outpatient basis. Before each chemotherapy cycle all patients underwent routine work-up including physical examination, blood tests and treatment-induced adverse effects assessment. IPCT regimens included modified FOLFOXIRI [27], GEMOX or GEMOXEL [28,29]. 

In the case of stable or responding disease after IPCT, three-dimensional conformal external beam radiotherapy (3D-RT) or an intensity-modulated technique (IMRT) were planned, given that both techniques seem to be equally effective and have a similar toxicity profile in the neoadjuvant setting [30]. The target volumes and organs at risk were contoured on each of the axial CT slices in the Helax-TMS treatment planning system (Nucletron Scandinavia, Uppsala, Sweden) or in the ADAC Pinnacle treatment planning system (Philips Radiation Oncology Systems, Fitchburg, WI, USA). The clinical target volume included the gross tumor volume of pancreas and the draining locoregional lymph nodes (peripancreatic and retroperitoneal). Conformation and field arrangement ensured that the organs at risk (kidneys, heart, liver, stomach, duodenum and spinal cord) received tolerable doses. Treatment planning followed International Commission on Radiation Units and Measurements recommendations. Patients were immobilized in the supine position. In general, four fields with 15-MV photons were employed to deliver 50 Gy over 4–5 weeks with conventional daily fractions of 1.8–2 Gy, 5 days per week. Five or seven coplanar, equally spaced beams were applied in a variable number of segments in IMRT plans. All patients received concurrent Capecitabine, 850 mg/m^2^ CORRECT bid during the days of radiation. Physical examination, blood test monitoring and therapy-induced toxicity were assessed on a weekly basis.

Surgery was scheduled 4–6 weeks after the end of the neoadjuvant protocol, once progressive disease was ruled out by preoperative CT-scan ± EUS.

### 2.3. Histological Data

A standardized histologic evaluation of the surgical specimen was performed in all resected patients. It included: a pathologic stage, vascular and perineural invasion assessment, lymph node status, lymph node ratio (LNR), resection margins (an R0 resection was defined as no tumor within 1 mm of the resection margin), a tumor regression grade according to the College of American Pathologists (CAP) grading system [31] (Table S2), and the degree of nodal response to treatment, evaluated with a 4-point scale adapted from the Miller & Payne grading system [32] (Table S3).

### 2.4. Postoperative Therapy and Follow Up

After surgery, adjuvant therapy was administered on a risk-adopted basis, according to pathological findings, patient characteristics and comorbidities. Patient follow-up was initially performed every 3–4 months for the first two years, every 6 months during years 3 and 4, and then annually. The surveillance protocol included physical examination, serum CA-19.9 level and CT-scan.

### 2.5. Toxicity

Toxicity during IPCT and CRT was evaluated and graded according to the National Cancer Institute Common Terminology for Adverse Events version 4.03 (NCI CTAE) scale. 

### 2.6. Statistical Analysis

For our first aim, descriptive and comparative statistical analyses were performed using SPSS statistical software (IBM SPSS Statistics, version 20, for Windows, Chicago Illinois, USA). Overall survival (OS) was defined as the time elapsed from diagnosis until death (all causes) or last contact when still alive. Progression-free survival (PFS) was calculated from the date of diagnosis to the date of progression (local and/or distant), death (all causes) or last contact when not relapsed. Overall survival and progression-free survival were determined by the Kaplan-Meier method and log-rank test. Two versions of the log-rank test were used (the Mantel-Cox and the Breslow method). All statistical tests were conducted at a two-sided significance level of 0.05.

This study was approved as an observational post-authorized study for medicines for human use, according to the Ethics and Clinical Investigation Committee of Navarra and the Drugs for Human Use Department of the Spanish Agency for Drugs and Health Products (AEMPS) (ethic code: CUN-QUI-2016-01, 23 March 2016).

### 2.7. Machine-Learning Algorithms

For our second aim, different machine-learning techniques were used to perform a predictive population model, including: Logistic Regression, Decision Tree, Random Forest, Support Vector Machine (SVM) and K-Nearest Neighbors Algorithm (KNN). A detailed description of the different techniques used is provided on Appendix A.

The goal of these algorithms is to provide an accurate prognostic information to resected PC patients treated with preoperative therapy. In order to avoid the learning process focusing excessively on the particular characteristics of our training data collected (overfitting), among approximately 140 clinical, pathological and analytical features from each patient, only those that were considered most influential in the individual risk of relapse after surgery were taken into account. A univariant exploratory analysis was performed with each of these initial features, and only 8 were finally selected. These selected variables were used for the training of each of the different machine-learning algorithms.

All the prediction models, except Random Forest, were validated using the 5-fold cross-validation technique, where the sample is divided into k sub-sets, in this case 5. This division is randomly performed, but always keeping the proportion of patients of each class in each of the subgroups. Once the sample is divided into subsets, k-1 subsets are taken as the training set and the remaining subgroup as the test set, in order to validate the algorithm. This process is repeated k times, allowing all possible combinations within the subsets. The result is the arithmetic mean obtained from the k repetitions. Random Forest was validated using a technique named bagging, which consists of bootstrapping the data and training each tree with one subset (bag). Then, each tree is validated with the instances out of its bag (OOB).

An external validation of the model was planned with a cohort of patients (20% of the global cohort) with potentially curable PC resected after a neoadjuvant approach at the Memorial Sloan Kettering Cancer Center (New York, NY, USA) between December 2008 and April 2016.

## 3. Results

### 3.1. Clinical Data

#### 3.1.1. Patients Characteristics

Baseline characteristics of the 40 resectable PC patients included in the retrospective analysis are summarized in Table 1. The median age at diagnosis was 63 (range 35 to 82) and the male/female ratio was 23/17. ECOG 0, 1 and 2 was found in 5 (12.5%), 33 (82.5%) and 2 (5%) patients, respectively. Most tumors were located in the head-isthmus of the pancreas (80%). According to EUS findings, T-stage was T2 in 3 patients (7.5%) and T3 in 37 patients (92.5%). Twenty-one patients (52.5%) were EUS-N0. Twenty-one of 40 patients (52.5%) required biliary stenting before receiving preoperative therapy.

#### 3.1.2. Multimodality Therapy Completion

**Figure 1:**
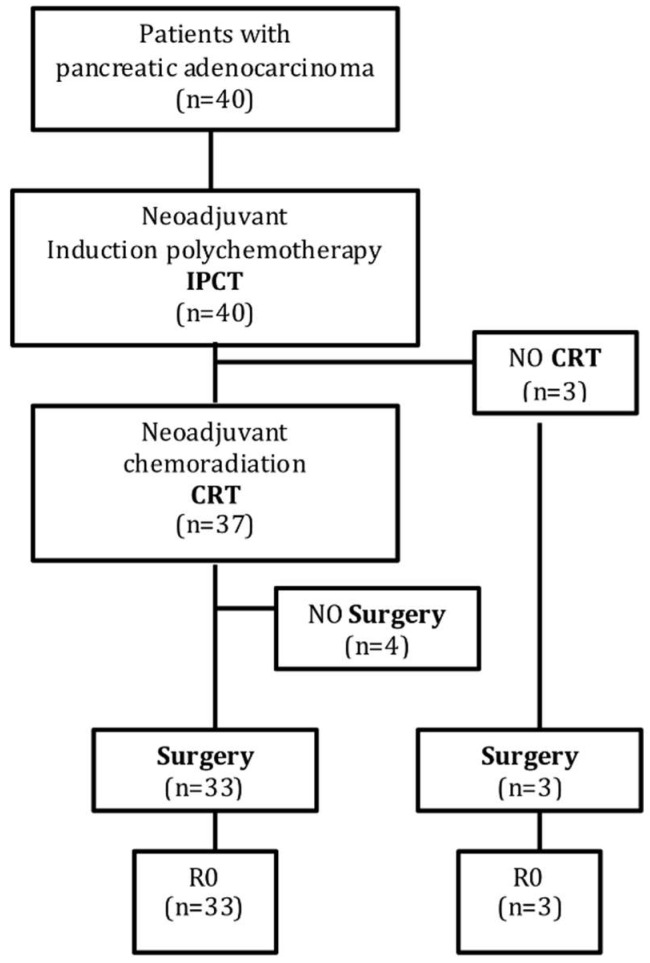
Patients flowchart through the therapeutic algorithm. IPCT: Induction polychemotherapy; CRT: Chemoradiation.

Figure 1 shows the patients flowchart through the therapeutic algorithm.

Induction polychemotherapy included mFOLFOXIRI (*n* = 14; 35%), GEMOXEL (*n* = 21, 52.5%) and GEMOX (*n* = 5, 12.5%). The median number of cycles administered was 4 (range 2 to 9). Three patients (7.5%) did not receive preoperative radiation therapy and were scheduled to surgery after IPCT due to recurrent episodes of cholangitis (one patient), necrotizing pancreatitis after 3 cycles of IPCT (one patient), and a suspicious resectable liver node detected in the post-IPCT re-staging (one patient). An R0 resection was achieved in all of them, including the patient with a new liver node that turned to be a gallbladder adenomyosis.

Thirty-seven patients (92.5%) received CRT after IPCT, 54% of them with 3D-RT and 46% with an IMRT technique. Median treatment length was 32 days (range 10 to 45).

Four patients (10%) could not be operated on after CRT. Three of them had a systemic relapse at the time of preoperative re-staging (development of liver metastases and peritoneal carcinomatosis in 2 and 1 patient, respectively). The remaining patient died 16 days after CRT due to a massive hematemesis. 

Overall, 36 patients (90%) underwent surgery: 33 patients after receiving the complete neoadjuvant schedule (IPCT + CRT), and 3 patients after being treated only with IPCT, as detailed above. The time frame from last neoadjuvant therapy administered (IPCT + CRT, or IPCT alone) to surgery ranged from 3.5 to 12.8 weeks.

After surgery, 9 patients (22.5%) received adjuvant treatment, most of them (77.8%) with the same protocol used in the neoadjuvant scenario. The median number of adjuvant cycles was 3 (range 1 to 4). One patient received adjuvant chemoradiation, because it had been omitted in the neoadjuvant setting. In this case, the patological report confirmed an R0 resection (minimal margin of 6 mm) and a ypT3N1 pancreatic adenocarcinoma with perineural invasion.

#### 3.1.3. Surgical Outcome and Pathological Results

Among the 36 patients who underwent surgery, 29 (80.6%) had a cephalic duodenopancreatectomy and 7 (19.4%) had a distal pancreatectomy. On an intent-to-treat basis, a R0 resection was achieved in 90% of the patients. CAP grade 0 was achieved in 5 patients (13.9%). Vascular and perineural invasion were observed in 3 (8.3%) and 10 (27.8%) patients, respectively. The median number of resected lymph nodes was 11 (range 2 to 22). Five patients (13.9%) were ypN+ (median number of affected nodes was 1). Grade A, B, and C nodal response was observed in 32 (88.9%), 1 (2.8%) and 3 (8.3%) patients. One patient had nodal involvement due to locorregional invasion, and was not classified according to a modified Miller & Payne lymph node grading system. No grade D response (complete nodal pathological response) was described in the pathological review of the resected specimens.

#### 3.1.4. Toxicity Profile

Toxicities associated with IPCT and CRT are described in Table 2.

During IPCT, all patients experienced at least one grade 1 or 2 adverse event. Sixteen patients (40%) had one or more grade 3–4 toxicities. Grade 3–4 toxicities included: neutropenia (10 patients, 25%), leukopenia (5 patients, 12.5%), cholangitis (5 patients, 12.5%), diarrhea (3 patients, 7.5%), asthenia (2 patients, 5%) and gastritis (1 patient, 2.5%). Up to 50% of grade 3–4 neutropenias were febrile neutropenias. Granulocyte colony-stimulating factors were used in 7 patients (17.5%). A dose reduction was required in 10 patients (25%), 9 of them in the group treated with mFOLFOXIRI and 1 in the Gemcitabine-based group. Ten patients (25%) had a treatment delay, 8 of them in the group treated with mFOLFOXIRI and 2 in the group treated with Gemcitabine-based chemotherapy. Thirteen patients (32.5%) required at least one hospital admission due to cholangitis (*n* = 5; 12.5%), diarrhea (*n* = 5; 12.5%: grade 2: *n* = 2; grade 3: *n* = 3)**,** febrile neutropenia (*n* = 4; 10%: grade 3: *n* = 3; grade 4: *n* = 1), atrial fibrillation (*n* = 2; 5%), pulmonary embolism (*n* = 1; 2.5%), perianal abscess (*n* = 1; 2.5%), and necrotizing pancreatitis (*n* = 1; 2.5%).

Grade 3-4 leukopenia, neutropenia and febrile neutropenia were more common in the group of patients treated with mFOLFOXIRI (35.7% vs. 0%; 57.1% vs. 7.7%; and 28.6% vs. 7.7%, respectively), whereas cholangitis was more frequent in the Gemcitabine-based group (15.4% vs. 7.1%). Cholangitis was only observed in patients with an endobiliary stent (23.8% vs. 0%), with a ratio of plastic vs. metallic stent in this group of patients of 3:2.

Overall, 11 patients (29.7%) had any grade 3 toxicity during CRT, including: cholangitis (5 patients, 13.5%), thrombocytopenia (4 patients, 10.8%) and asthenia (2 patients, 5.4%). No grade 4 toxicity associated to CRT was reported. Six patients (16.2%) required at least one hospital admission due to cholangitis (*n* = 5; 13.5%), grade 1 fever (*n* = 1; 2.7%), grade 2 nausea (*n* = 1; 2.7%), and grade 3 asthenia (*n* = 1; 2.7%). One patient required CRT discontinuation due to cholangitis. Besides, 6 patients (16.2%) required concomitant capecitabine dose reduction.

There were no perioperative or in-hospital deaths related to the surgical procedure. Eight patients (22.2%) developed one or more early postoperative complications, including: paralytic ileus (*n* = 4; 11%), infected pancreatic fistula (*n* = 2; 5.6%), respiratory distress syndrome (*n* = 1; 2.8%), gastro-jejunal anastomosis bleeding (*n* = 1; 2.8%), hemoperitoneum secondary to pancreatic-duodenal artery bleeding (*n* = 1; 2.8%), left renal fossa hematoma (*n* = 1; 2.8%) and abdominal sepsis (*n* = 1; 2.8%). In the last 3 cases, an urgent exploratory laparotomy was needed, with a later ICU observation period. The median length of hospitalization was 8.36 days (range 4–35). Ten patients (27.8%) required a red blood cell transfusion.

Nine patients (25%) had late surgical complications, including: lymphocele (*n* = 2; 5.6%), peripancreatic collection, (*n* = 2; 5.6%), perihepatic collection (*n* = 1; 2.8%), gastric erosion bleeding (*n* = 1; 2.8%), splenectomy surgical site hematoma (*n* = 1; 2.8%), paralytic ileus (*n* = 1; 2.8%), inflammatory-infectious process (*n* = 1; 2.8%), antrum-pyloric stenosis, that required a surgical derivation (*n* = 1; 2.8%), and pancreatectomy site pseudo-cyst (*n* = 1; 2.8%). All these complications were uneventfully managed.

#### 3.1.5. Patients Long-Term Outcome

**Figure 2:**
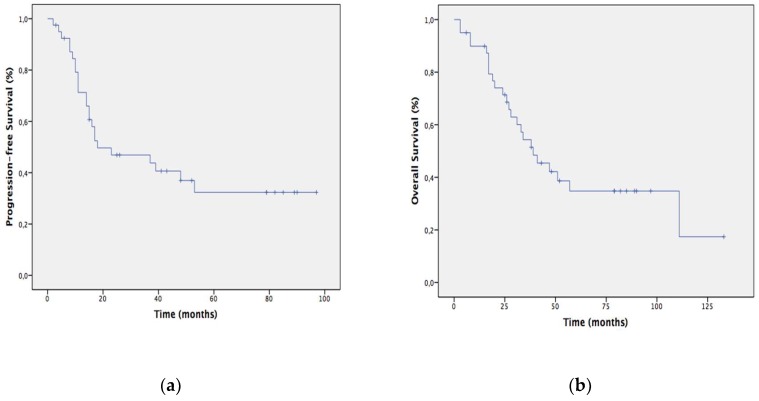
Kaplan-Meier estimates of progression-free survival (**a**) and overall survival (**b**) among patient with resectable PC treated with the neoadyuvant approach.

After a median follow-up of 33.5 months (range 3 to 133 months), median progression-free survival (PFS) was 18 months and median overall survival (OS) was 39 months (Figure 2). The 1, 2, 3 and 5-year actuarial PFS were 71.3%, 46.9%, 43.8% and 32.3% respectively. The 1, 2, 3 and 5-year actuarial OS were 89.9%, 71.4%, 51.5% and 34.8% respectively. Median PFS (37 months vs. 18 months; *p* = 0.026) and OS (47 months vs. 8 months; *p* = 0.003) were significantly longer in those patients able to complete the whole therapeutic program (IPCT, CRT and surgery), compared to those who did not receive CRT or surgery. The discrepancy between OS and PFS in this last group of patients may be explained by the reported death due to massive hematemesis before surgery, without evidence of relapse.

Surgical margins status and the presence of vascular invasion among ypN0 patients significantly correlated with survival outcomes. Among those patients who completed the whole therapeutic program, the median PFS was 37 months for those receiving FOLFOXIRI compared to 17 months for those receiving gemcitabine-based IPCT, with a 3-year PFS of 62.3% and 45.5%, respectively.

Twenty-four patients (60%) have relapsed. The pattern of relapse was distant in 18 cases (75%), locoregional in 1 case (4.2%), and both, local and distant in 5 cases (20.8%). Liver was the most common site for distant progression (27.5%). Twenty of the relapsed patients (83.3%) underwent a second-line treatment. Among them, 17 died due to disease progression, 1 died due to cardiological comorbidity and 2 patients were alive at the end of the follow-up. 

### 3.2. Predictive Population Model 

#### 3.2.1. Model Development

Baseline characteristics of the 45 operated patients (resectable/borderline resectable; 37/8) included in the machine-learning algorithms are summarized in Table 3. Up to 42 (93.3%) of resected PC received multimodal neoadjuvant therapy (IPCT + CRT). Thirty-seven patients (82.2%) had a cephalic duodenopancreatectomy and 8 patients (17.8%) had a distal pancreatectomy. Only 28.9% of patients (*n* = 13) received adjuvant treatment. Thirty patients (66.7%) relapsed, most of them at distant sites (*n* = 21; 72.4%).

Several known prognostic features related to the risk of relapse were collected and are summarized in Table 4. By univariate analysis, the most relevant for the model were selected, including: ECOG, the type of IPCT employed, the use of granulocyte-colony-stimulating factors, the type of surgery, the number of resected lymph nodes, the modified LNR, the presence of perineural invasion and the surgical margins status.

**Figure 3:**
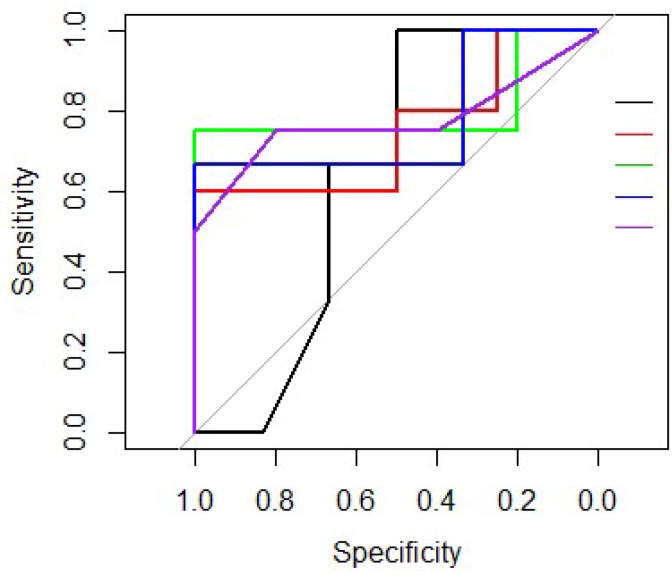
Receiver Operating Characteristic (ROC) curves of Logistic Regression predictive population model for each of the 5-fold cross validation. Each ROC curve is represented in a different color (black, red, green, blue and purple).

Each of the different machine-learning algorithms previously described were trained with the 8 selected variables (Table 5). After applying a 5-fold cross-validation technique, it was concluded that Logistic Regression was the best predictive algorithm. According to the three intermediate steps (step Akaike criterion (AIC), non-linear trends and interactions), the appropriate components that should be retained in the model were: the use of granulocyte colony-stimulating factors (yes/no), the number of resected lymph nodes, the presence of perineural invasion (yes/no) and the surgical margins status (R0/R1). Taking into account these features, the model predicts the probability of relapse at 2 years after surgery for an individual patient with an accuracy of 0.71 (95% IC 0.56–0.84, *p* = 0.005), a sensitivity of 0.70, a specificity of 0.73 and a mean area under the curve (AUC) of 0.75 (Table 5 and Figure 3).

#### 3.2.2. External Validation of the Model

An external validation of the model was performed with a cohort of PC patients from a USA institution. 

The external cohort comprised 49 PC patients, but only 33 of them were considered resectable or borderline-resectable at diagnosis. Among them, in only 27 patients were the required components of the model available. After a descriptive analysis of the cohort, a higher median number of resected lymph nodes was reported in the validation cohort (mean 24.37; median 21; minimum 9; maximum 44) compared to the training dataset (mean 10.93; median 9; minimum 2; maximum 27). In order to minimize differences related to the extent of the lymphadenectomy, only 11 patients from the validation dataset with a number of resected lymph nodes up to the 90th percentile of the training dataset were included in the validation cohort. The descriptive analysis of the four components in the validation dataset is summarized in Table 6. Among the 11 patients from the validation cohort, the model-predicted outcome matched with the observed outcome in 7 patients. The predictive accuracy of the model at the individual level was 64%.

## 4. Discussion

Several studies have shown the feasibility and activity of a preoperative treatment in potentially curable PC, [14,33,34,35,36,37,38,39,40,41,42,43,44] (Table 7), with R0 resection rates ranging from 25% [36] to 76% [14], and median overall survival times between 9.4 [40] and 34 [14] months, with no increase in the rates of postoperative complications [45]. Recently a propensity score-matched analysis has suggested a survival benefit in favor of neoadjuvant therapy compared to upfront surgery alone (HR 0.72) or followed by adjuvant treatment (HR 0.83) [46], although no randomized phase III trials have been reported to date.

In the present study we combine the use of intensive IPCT and preoperative CRT in a subset of patients with potentially curable PC. In the metastatic setting, triplet regimens have usually correlated with improved efficacy, [47,48,49,50] and this has encouraged its application in locally advanced disease. A preliminary pilot trial with neoadjuvant mFOLFOXIRI in resectable PC has also shown that this regimen is feasible and tolerable in this setting [14]. The preoperative use of radiotherapy in resectable PC aims at improving local control [43,44], R0 resection rates [51] and reducing the incidence of postsurgical complications, due to the induction of fibrosis in pancreatic tissue and surgical bed. Indeed, preoperative radiotherapy improves suitability of pancreatic tissue for anastomosis and reduces the risk of developing a pancreatic fistula or anastomotic leak [43,52,53]. Preliminary data have shown that preoperative chemoradiotherapy significantly improves outcomes in resectable PC compared to immediate surgery [13].

With a R0 resection rate of 90% and a median overall survival of 39 months, our results overlap with those achieved with similar strategies [14,34,44] (Table 6). Our data seem especially noteworthy in the subgroup of resected patients who completed the whole neoadjuvant program, with a median PFS of 37 months and a median OS of 47 months. Local recurrence as the only pattern of relapse was identified in 4.2%, which is a low result for outcomes reported with preoperative strategies. 

The use of an intensified neoadjuvant approach is not without cost. In our series, up to 40% of patients developed grade 3–4 toxicities due to IPCT, 25% required dose reductions or treatment delays (most of them in the mFOLFOXIRI group), and the hospitalization rate was 32.5%. An additional 30% of patients had grade 3 adverse events related to CRT. Our cholangitis rate (12.5% with IPCT and 13.5% with CRT) is in the range of that observed in other studies [42], and clearly related to biliary stents, as described previously [54,55].

Considering the high rate of distant relapse in our series, and in an attempt to identify the subset of operated patients with a higher likelihood of relapse, we aim to build up a predictive model based on supervised machine-learning algorithms. The prognostic impact of the four features included in the final prediction model has been previously described on an individual basis. [56,57,58,59,60,61] Of note is the role of granulocyte colony-stimulating factors, whose impact on cancer survival has been previously reported in solid tumors. [62] The importance of increasing chemotherapy relative dose intensity has also been described in PC patients, with preliminary encouraging results. [63,64] With an accuracy above 60% for a 2-year relapse-risk after surgery, this model may be a useful tool, with clinical practice decision-making implications. Among at high risk patients, more intensive surveillance, the use of adjuvant treatment, or even the inclusion of these patients into clinical trials may be considered.

Our study has several limitations, including: its retrospective nature (with the inherent biases related to this type of studies), the small sample size, the heterogeneity in the IPCT and radiotherapy techniques applied, and the fact of being a single institutional experience. Additionally, the limited number of patients included in the dataset of our second aim reduce the accuracy for quantifying interpatient variability effects. In addition, no pharmacodynamic information such as tumor size was included in the prediction model, due to the difficult examination of tumor downstaging by radiologic procedures because of intense inflammation and fibrosis induced by neoadjuvant treatment.

## 5. Conclusions

In summary, the use of an intensified preoperative program with IPCT followed by CRT offers encouraging results in terms of R0 resection rates and survival times at an expense of manageable toxicity. Implementation of machine-learning algorithms may help to identify at-risk patients and tailor adjuvant strategies.

## Figures and Tables

**Table 1:** Baseline characteristics of patients with resectable PC treated with a multimodality neoadjuvant approach, *n* = 40.

Variables	*n* (%)
**Age-years**	
Median	63
Range	35–82
**Gender**	
Male	23 (57.5)
Female	17 (42.5)
**ECOG**	
0	5 (12.5)
1	33 (82.5)
2	2 (5)
**Location**	
Head-Isthmus	32 (80)
Body-Tail	8 (20)
**Baseline EUS-T stage**	
T1-T2	3 (7.5)
T3	37 (92.5)
**Baseline EUS-N stage**	
N0	21 (52.5)
N+	11 (27.5)
Nx	8 (20)
**Biliary stent**	
No	19 (47.5)
Yes	21 (52.5)
**Neoadjuvant chemotherapy regimen**	
Gemcitabine-based	26 (65)
mFOLFOXIRI	14 (35)
**Neoadjuvant radiotherapy technique**	
3D-RT	20 (54.1)
IMRT	17 (45.9)

**Table 2:** Adverse events during neoadjuvant regimen.

Adverse Event	IPCT ^1^ (*n* = 40)			CRT ^2^ (*n* = 37)		
Type	Grade 1/2 *n* (%)	Grade 3 *n* (%)	Grade 4 *n* (%)	Grade 1/2 *n* (%)	Grade 3 *n* (%)	Grade 4 *n* (%)
**Hematological**						
Anemia	37 (92.5)	-	-	33 (89.2)	-	-
Leukopenia	19 (47.5)	2 (5)	3 (7.5)	29 (78.4)	-	-
Neutropenia	7 (17.5)	9 (22.5)	1 (2.5)	8 (21.6)	-	-
Febrile neutropenia	1 (2.5)	4 (10)	1 (2.5)	1 (2.7)	-	-
Thrombocytopenia	26 (65)	-	-	27 (73)	4 (10.8)	-
**Non-hematological**						
Nausea/Vomiting	12 (30)	-	-	15 (40.5)	-	-
Anorexia	18 (45)	-	-	20 (54)	-	-
Diarrhea	15 (37.5)	3 (7.5)	-	6 (16.2)	-	-
Gastritis	7 (17.5)	1 (2.5)	-	13 (35.1)	-	-
Mucositis	10 (25)	-	-	2 (5.4)	-	-
Asthenia	32 (80)	2 (5)	-	21 (56.7)	2 (5.4)	-
Peripheral neuropathy	22 (55)	-	-	6 (16.2)	-	-
Hand-foot syndrome	1 (2.5)	-	-	1 (2.7)	-	-
Cholangitis	-	5 (12.5)	-	-	5 (13.5)	-

Abbreviations: ^1^ IPCT (induction polychemotherapy); ^2^ CRT (Chemoradiotherapy).

**Table 3:** Baseline characteristics of patients included in 2-year relapse risk prediction model, *n* = 45.

Variables	*n* (%)
Age-years	
Median	64
Range	44-80
Gender	
Male	23 (51.1)
Female	22 (48.9)
ECOG	
0	8 (17.8)
1	35 (77.8)
2	2 (4.4)
Location	
Head-Isthmus	34 (75.6)
Body-Tail	10 (22.2)
Multifocal	1 (2.2)
Baseline EUS-T stage	
T1-T2	4 (8.9)
T3	40 (88.9)
T4	1 (2.2)
Baseline EUS-N stage	
N0	27 (60)
N+	11 (24.4)
Nx	7 (15.6)
Resectability	
Resectable	37 (82.2)
Borderline-resectable	8 (17.8)
Neoadjuvant approach	
IPCT + CRT	42 (93.3)
IPCT	3 (6.7)
Duration of IPCT-days	
Median	53
Range	40-125
Number of CRT session	
Median	25
Range	18-30
Type of surgery	
Cephalic duodenopancreatectomy	37 (82.2)
Distal pancreatectomy	8 (17.8)
Adjuvant treatment	
Yes	13 (28.9)
No	32 (71.1)
Relapse	
Yes	30 (66.7)
No	15 (33.3)
Relapse at 2 years	
Yes	22 (48.9)
No	23 (51.1)
Type of relapse	
Local	2 (6.7)
Distant	22 (73.3)
Local and distant	6 (20)

ECOG: Eastern Cooperative Oncology Group; EUS: Endoscopic ultrasound

**Table 4:** Features included in the preliminary analysis for the machine learning algorithms.

Variables	*n* (%)
Gender	
Male	23 (51.1)
Female	22 (48.9)
Age-years	
Min.	44
Median	64
Mean	63
Max.	80
Resectability	
Resectable	37 (82.2)
Borderline-resectable	8 (17.8)
ECOG	
0	8 (17.8)
1	35 (77.8)
2	2 (4.4)
Neoadjuvant chemotherapy regimen	
mFOLFOXIRI	18 (40)
Gemcitabine-based	27 (60)
Granulocyte colony-stimulating factors	
No	37 (82.2)
Yes	8 (17.8)
Neoadjuvant radiotherapy technique	
3D-RT	18 (40)
IMRT	21 (46.7)
Not reported	6 (13.3)
Type of surgery	
Cephalic duodenopancreatectomy	37 (82.2)
Distal pancreatectomy	8 (17.8)
ypT	
ypT0	6 (13.3)
ypT1	19 (42.2)
ypT2	6 (13.3)
ypT3	12 (26.7)
ypTx	2 (4.4)
ypN	
ypN0	41 (91.1)
ypN1	4 (8.9)
CAP	
0	6 (13.3)
1	23 (51.1)
2	10 (22.2)
3	6 (13.3)
CAP Group	
CAP 0–1	29 (64.4)
CAP 2–3	16 (35.6)
Pathological complete response	
No	37 (82.2)
Yes	8 (17.8)
Resected lymph nodes	
Min.	2
Median	9
Mean	10.93
Max.	27
Pathological lymph nodes	
Min.	0.00
Median	0.00
Mean	0.16
Max.	3.00
Modified LNR: 1 + *n*° pathological/1 + *n*° resected	
Min.	0.036
Median	0.100
Mean	0.125
Max.	0.333
Miller & Payne nodal response	
A	41 (91.1)
B	1 (2.2)
C	3 (6.7)
D	0 (0)
Vascular invasion	
No	40 (88.9)
Yes	5 (11.1%)
Perineural invasion	
No	31 (68.9)
Yes	14 (31.1)
Surgical margins	
R0 (Not involved, >1 mm)	43 (95.6)
R1 (Involved, <1 mm)	2 (4.4)
SCORE ^1^	
1	6 (13.3)
2	25 (55.6)
3	14 (31.1)
Dose intensity > 80% ^2^	
No	12 (27.3)
Yes	32 (72.7)
Progression disease at 2 years	
No	23 (51.1)
Yes	22 (48.9)

^1^ SCORE include 6 variables (CAP = 0, ypT0-N0, A or D nodal response according to Miller & Payne grading system, absence of vascular invasion, absence of perineural invasion, and R0 resection). SCORE 1 include 6/6 variables, SCORE 2 include 4–5/6 variables, and SCORE 3 include ≤3/6 variables. ^2^ Data not collected in one patient, who received some of neoadjuvant chemotherapy cycles in a different institution.

**Table 5:** Results from different machine-learning algorithms.

Variable	Logistic Regression	Decision Tree	Random Forest	Support Vector Machine	K-Nearest Neighbours
**Accuracy**	0.71	0.60	0.67	0.60	0.58
**Sensitivity**	0.70	0.83	0.74	0.65	0.65
**Specificity**	0.73	0.36	0.59	0.55	0.50
**PPV ^1^**	0.73	0.58	0.65	0.60	0.58
**NPV ^2^**	0.70	0.67	0.68	0.60	0.58
**AUC**	0.75	0.61	0.67	0.61	0.58

Abbreviations: ^1^ PPV (Positive predictive value); ^2^ NPV (Negative predictive value).

**Table 6:** Baseline features of the validation dataset that were included in the predictive model.

Variables	*n* (%)
Granulocyte colony-stimulating factors	
No	4 (36.4)
Yes	7 (63.6)
Perineural invasion	
No	2 (18.2)
Yes	9 (81.8)
Surgical margins	
R0 (Not involved)	8 (72.7)
R1 (Involved)	3 (27.3)
Resected lymph nodes	
Min.	9
Median	17
Mean	15.55
Max.	19
Progression disease at 2 years	
No	4 (36.4)
Yes	7 (63.6)

**Table 7:** Neoadjuvant studies in resectable pancreatic cancer.

Study	NA	ChT	RT	CRT	*n*	Stage	Resection	R0 Rate G/Res	Median OS (Months)			Survival Rate (%)		
									G	Res	NRes	G	Res	NRes
Ishikawa (1994) [33]	RT	-	50 Gy	-	54	R	74%	-	15	-	9	30 (2y), 22 (5y)	28 (3y), 22 (5y)	17 (1y), 0 (2y)
Evans (1992) [37]	CRT	-	-	5-FU, 50.4Gy	28	R	61%	50%/82%	-	-	-	-	-	-
Evans (2008) [38]	CRT	-	-	Gemcitabine 30 Gy	86	R	74%	66%/95%	22.7	34	7	27 (5y)	36 (5y)	0 (5y)
						BR								
Turrini (2009) [39]	CRT	-	-	5-FU Cisplatin 45 Gy	102	R	61%	56%/92%	17	23	11	10 (5y)	18 (5y)	0 (5y)
Le Scodan (2009) [40]	CRT	-	-	5-FU Cisplatin 50 Gy	41	R	63%	51%/80.7%	9.4	11.7	5.7	41 (1y), 20 (2y)	48 (1y), 32 (2y)	40 (1y), 0 (2y)
Kim (2013) [41]	CRT	-	-	Gemcitabine	68	R	63%	53%/84%	18.2	27.1	10.9	62 (1y), 44 (2y)	82 (1y), 62 (2y)	33 (1y), 17 (2y)
				Oxaliplatin		BR								
				30Gy		I								
Golcher (2015) [42]	CRT	-	-	Gemcitabine Cisplatin 55.8 to 50.4Gy	66	R	58%	51%/89%	17.4	25	-	39 (2y), 12 (3y)	-	-
						BR								
Casadei (2015) [43]	ChT-CRT	Gemcitabine	-	Gemcitabine 54 Gy	38	R	61.1%	38.9%/64%	22.4	-	-	-	-	-
Varadhachary (2008) [44]	ChT-CRT	Gemcitabine Cisplatin	-	Gemcitabine 30 Gy	90	R	58%	55%/96%	17.4	31	10.5	37 (2y), 19 (4y)	60 (2y), 36 (4y)	-
						BR								
O’Reilly (2014) [34]	ChT	Gemcitabine Oxaliplatin	-	-	38	BR	71%	53%/74%	27.2	NR	15	63 (18m)	78 (18m)	25 (18 m)
Heinrich (2008) [35]	ChT	Gemcitabine Cisplatin	-	-	28	R	89%	71%/80%	26.5	19.1	-	-	-	-
Palmer (2007) [36]	ChT	Gemcitabine Cisplatin Vs. Gemcitabine alone	-	-	50	R	70% Vs. 38%	46%/75% Vs. 25%/75%	15.6 Vs. 9.9	28.4 (global)	-	62 (1y) Vs. 42 (1y)	77.8 (global)	-
De W Marsh (2017) [14]	ChT	mFOLFIRINOX	-	-	21	R	81%	76%/94%	34	35.5	10.1	80 (1y), 60(2y)	81 (1y), 71 (2y)	33 (1y), 0 (2y)

Abbreviations: NA (Neoadjuvant Approach); ChT (Chemotherapy); RT (Radiotherapy); CRT (Chemoradiotherapy); ChT-CRT (Chemotherapy followed by Chemoradiotherapy); N (number of patients included in the study); R (resectable PC); BR (borderline-resectable PC); I (Irresectable PC); NR (Not Reached); G (Global); Res (Resected); NRes (Non-resected).

## Supplementary Materials

The following are available online at https://www.mdpi.com/2072-6694/11/5/606/s1, Table S1: Resectability criteria by National Comprehensive Cancer Network, Table S2: Tumor regression grade according to the College of American Pathologists (CAP) grading system, Table S3: Grade of nodal treatment response scale adapted from the Miller & Payne grading system.

## Appendix A. Different Machine-Learning Techniques Used to Perform a Predictive Population Model

1. Logistic Regression:

It predicts the presence or absence of a characteristic or result according to the values of a set of predictors. It is similar to a linear regression model but is adapted for models in which the dependent variable is dichotomous. The logistic regression coefficients can be used to estimate the odds ratio of each variable independent of the model. To obtain the best classifier we perform three steps. First, we apply Step AIC criterion to obtain the best model with the optimal combination of features. Second, we analyse if any of the selected features present non-linear tendencies by a visual inspection. Last, we consider interactions between the features and we implement Step AIC criterion again to obtain the best predictor.

2. Decision Tree:

It is a map of the possible outcomes of a series of related decisions. It starts with a single node and then branches into possible outcomes. Each of those results creates additional nodes, which branch out into other possibilities. This gives it a shape similar to that of a tree. When a new instance is to be classified, its characteristics are analysed according to the decision tree structure, which indicates the outcome to that instance with the probability to belonging to that category.

3. Random Forest:

It is a decision tree ensemble that combines the knowledge generated by a collection of individual tree making use of randomness in the process. In order to classify a new patient, each tree provides a classification, which its corresponding probability, and the category with the highest probability is chosen.

4. Support Vector Machine (SVM):

This algorithm learns the decision boundary of two different classes of entry points. When the points are non-linearly separable, the algorithm transforms the domain to a higher dimensional space where they can be separated by a linear hyperplane. Moreover, the decision boundary is optimized so that the hyperplane is equidistant to the nearest instances of each category.

5. K-Nearest Neighbours Algorithm (KNN):

This algorithm consists in predicting the category of an instance based on the categories of the closets instances. The proximity of two instances is measured by the similarity of each of their features using for example the Euclidean distance.
