# Supplementary material for: Use of Machine-Learning Algorithms in Intensified Preoperative Therapy of Pancreatic Cancer to Predict Individual Risk of Relapse
Source: Cancers (Basel). 2019 Apr 30;11(5):606. doi: 10.3390/cancers11050606 (PMC6562932; doi:10.3390/cancers11050606)
Supplement: Supplementary file 1 [file cancers-11-00606-s001.pdf]

Supplementary Materials: Use of Machine-Learning Algorithms in Intensified Preoperative Therapy of Pancreatic Cancer to Predict Individual Risk of Relapse

Pablo Sala Elarre, Esther Oyaga-Iriarte, Kenneth Yu, Vicky Baudin, Leire Arbea Moreno, Omar Carranza, Ana Chopitea Ortega, Mariano Ponz-Sarvise, Luis D Mejías Sosa, Fernando Rotellar Sastre, Blanca Larrea Leoz, Yohana Iragorri Barberena, Jose C Subtil Iñigo, Alberto Benito Boíllos, Fernando Pardo and Javier Rodríguez Rodríguez

Table S1. Resectability criteria by National Comprehensive Cancer Network.

Resectability Group	Arterial	Venous
**Resectable**	No tumor contact with celiac axis (CA), superior mesenteric artery (SMA) or common hepatic artery (CHA).	No tumor contact with superior mesenteric vein (SMV) or portal vein (PV).
		Tumor contact with SMV or PV ≤ 180° without vein contour irregularity
**Borderline-Resectable**	Pancreatic head/uncinate process:<br>Tumor contact with CHA without extension to CA or hepatic artery bifurcation.<br>Tumor contact with SMA ≤ 180°<br>Tumor contact with variant arterial anatomy that may affect surgical planning.	Tumor contact with SMV or PV < 180° with vein contour irregularity or thrombosis of the vein but with suitable vessel proximal and distal to the site of involvement.
	Pancreatic body/tail:<br>Tumor contact with CA ≤ 180°<br>Tumor contact with CA >180º without involvement of the aorta and gastroduodenal artery (permitting a modified Appleby procedure).	Tumor contact with SMV or PV > 180°
		Tumor contact with inferior vena cava (IVC).

Table S2. Tumor regression grade according to the College of American Pathologists (CAP) grading system.

CAP Grade	Definition
0	No viable residual tumor (pathologic complete response (pCR))
1	Minimal residual cancer with single cells or small groups of cancer cells (marked response)
2	Residual cancer outgrown by fibrosis (moderate response)
3	Extensive residual cancer (poor or no response)

Table S3. Grade of nodal treatment response scale adapted from the Miller & Payne grading system.

Miller & Payne Grade	Definition
A	True negative lymph nodes, with no evidence of pathological effects related to treatment
B	Infiltrated lymph nodes, with no evidence of pathological response to treatment
C	Infiltrated lymph nodes, with partial regression due to neoadjuvant therapy
D	Complete pathological response in a previously infiltrated lymph node

© 2019 by the authors. Licensee MDPI, Basel, Switzerland. This article is an open access article distributed under the terms and conditions of the Creative Commons Attribution (CC BY) license (http://creativecommons.org/licenses/by/4.0/).
